# Carbapenem resistance in *Escherichia marmotae*: characterization of IncL plasmids carrying *bla*_OXA-48_

**DOI:** 10.1093/jac/dkaf188

**Published:** 2025-06-16

**Authors:** Astrid Rasmussen, Anette M Hammerum, Frank Hansen, Lillian M Søes, Michael Pedersen, Hans L Nielsen, Flemming Scheutz, Henrik Hasman, Louise Roer

**Affiliations:** Department of Bacteria, Parasites and Fungi, Statens Serum Institut, Copenhagen, Denmark; Department of Bacteria, Parasites and Fungi, Statens Serum Institut, Copenhagen, Denmark; Department of Bacteria, Parasites and Fungi, Statens Serum Institut, Copenhagen, Denmark; Department of Clinical Microbiology, Copenhagen University Hospital—Amager and Hvidovre, Hvidovre, Denmark; Department of Clinical Microbiology, Copenhagen University Hospital—Amager and Hvidovre, Hvidovre, Denmark; Department of Clinical Microbiology, Aalborg University Hospital, Aalborg, Denmark; Department of Clinical Medicine, Aalborg University, Aalborg, Denmark; Department of Bacteria, Parasites and Fungi, Statens Serum Institut, Copenhagen, Denmark; Department of Bacteria, Parasites and Fungi, Statens Serum Institut, Copenhagen, Denmark; Department of Bacteria, Parasites and Fungi, Statens Serum Institut, Copenhagen, Denmark

Dear Sir,

Carbapenemase-producing *Enterobacterales* (CPE) represent a significant concern to human health. Among these, the *bla*_OXA-48_ gene, often located on the highly stable incompatibility group L (IncL) plasmids. These plasmids enable rapid horizonal gene transfer across bacterial species, posing an emerging threat.

*Escherichia marmotae* was first described in 2015. It was initially considered susceptible to antimicrobials. However, present-day findings indicate resistance to last-resort agents and high pathogenic potential. The genetic proximity to *E. coli* raises concerns about its potential in AMR dissemination. A recent study identified *E. marmotae* isolates harbouring extended-spectrum β-lactamase (ESBL) genes located on plasmids, suggesting the capacity to acquire and propagate AMR via horizontal gene transfer.^1^ Sivertsen *et al.* reported *bla*_KPC_-carrying *E. marmotae*, reinforcing its invasive pathogen potential,^2^ aligning with findings by Liu *et al*.^3^

Since 2014, CPE samples have been collected and whole genomes sequenced as part of the Danish CPO surveillance programme. In 2023 and 2024, two carbapenemase-producing *E. marmotae* isolates were submitted to the National Reference Laboratory at Statens Serum Institut (NRL, SSI) for verification and characterization. Whole-genome sequencing using short- and long-read technologies identified resistance genes, virulence factors, plasmid content and assessed genetic relatedness. The *bla*_OXA-48_-carrying IncL plasmids were compared for structural similarities.

The two *E. marmotae* isolates originated from geographically distinct patients. The first isolate, submitted in 2023 by Aalborg University Hospital, was confirmed as *E. marmotae* carrying *bla*_OXA-48_. Retrospective data revealed that Patient 1 had a previous infection with *bla*_OXA-48_-producing *K. oxytoca* 6 months earlier. In the *E. marmotae*, virulence factors *chuA*, *iss*, *ompT* and *sitA* were identified.

In 2024, a second *E. marmotae* isolate was submitted by Copenhagen University Hospital, Amager and Hvidovre. This isolate also carried *bla*_OXA-48_ and a broad array of virulence genes, including the porcine colonization factor F4 (*faeD*/*E*/*F*/*H*/*I*) typical of enterotoxigenetic *E. coli* (ETEC), ExPEC associated genes *(papAH/C* and *kpsMII*) and genes characteristic of hybrid ExPEC-STEC O80:H2 strains (*cia*, *etsC*, *iss*, *traJ*, *ompT*, *hlyF*, *iroN*).^4^ The presence of haemolysin (*hlyF*) and haem uptake system (*chuA*) suggest additional virulence factors enhancing bacterial survival and pathogenicity. Retrospective searches revealed that Patient 2 had a previous carbapenemase-negative *E. marmotae*, which was neither collected nor sequenced.

*E. marmotae* has often been misidentified as *E. coli*, partly because it was not included in the Bruker^™^ MALDI-TOF database until 2020. Consequently, a retrospective rMLST analysis of 907 Danish CPE *E. coli* isolates was performed, but no additional *E. marmotae* cases were identified.

Although reports of resistant *E. marmotae* remain limited, Sivertsen *et al.* found *bla*_KPC_ in one and *bla*_CTX-M_ in two out of 45 *E. marmotae* genomes.^2^ By mid-2024, 259 *E. marmotae* genomes were available in the NCBI database, with 23 (9%) carrying ESBL (*bla*_CTX-M-14_; *n* = 10, *bla*_CTX-M-15_; *n* = 2, *bla*_CTX-M-1_; *n* = 1, *bla*_CTX-M-32_; *n* = 1), pAmpC (*bla*_CMY-2_; *n* = 6) or carbapenemase genes (*bla*_KPC-2_; *n* = 1, *bla*_KPC-3_; *n* = 1), whereas one isolate carried both *bla*_CTX-M-14_ and *bla*_CMY-2_. Thus, the two Danish isolates represent, to our knowledge, the first reported cases of OXA-48 producing *E. marmotae*.

Genomic comparison revealed 28 757 SNP differences between the two Danish *E. marmotae* isolates. They did not show close genetic relatedness to any of the 257 publicly available *E. marmotae* genomes. The closest matches were 163 and 251 SNPs apart, respectively, suggesting two independent acquisitions. Epidemiological investigations found no links between the patients, further supporting this conclusion.

**Figure 1.**
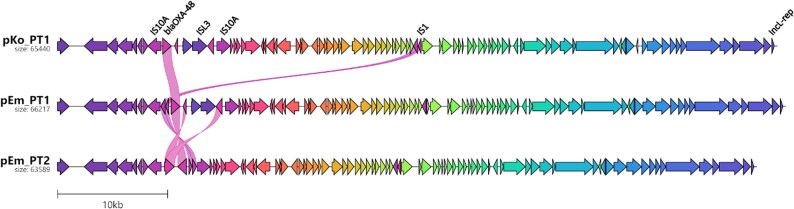
Comparison of three IncL plasmids carrying *bla*_OXA-48_, from two Danish patients. pKo_PT1 reconstructed from a *Klebsiella oxytoca* and pEm_PT1 from an *Escherichia marmotae* from Patient 1. pEm_PT2 from an *E. marmotae* from Patient 2.

Both *E. marmotae*, as well as the earlier *K. oxytoca* from Patient 1, carried IncL plasmids associated with *bla*_OXA-48_.^5,6^ Genomes annotated with Bakta from long-read assemblies and visualized with Clinker^7^ confirmed similar plasmid backbones (Figure 1). Using Pling,^8^ structural comparison of the *E. marmotae* plasmid from Patient 1 (pEm_PT1) and the *K. oxytoca* plasmid (pKo_PT1) revealed a single rearrangement; an IS1 insertion upstream of *bla*_OXA-48_ gene (Figure 1). Such rearrangements are common in interspecies plasmid transmissions, suggesting plasmid transfer between the *K. oxytoca* isolate and the *E. marmotae* isolate in Patient 1.

Comparison of pEm_PT1 from Patient 1 and the plasmid from Patient 2 (pEm_PT2) identified two additional rearrangements; an inversion between IS10A elements and the insert/deletion of a KilA-N domain protein and ISL3 insertion element downstream of *bla*_OXA-48_. Despite structural similarity of the two plasmids, no epidemiological links were found between the two patients. With IncL plasmids carrying *bla*_OXA-48_ now near endemic status, the plasmids were probably acquired from another bacterial host.

In conclusion, *E. marmotae*, traditionally considered susceptible, has now been shown to acquire *bla*_OXA-48_-carrying IncL plasmids. Here we report two independent acquisition events, adding *E. marmotae* to the list of potential recipients of this worldwide disseminated plasmid. The combination of hybrid ETEC-ExPEC virulence traits and resistance potential of *E. marmotae*, reinforces the importance of continued CPE surveillance and genomic characterization at both species and plasmid level.
